# Supplementary data for the biological age linked to oxidative stress modifies breast cancer aggressiveness

**DOI:** 10.1016/j.dib.2018.03.132

**Published:** 2018-04-03

**Authors:** María del Mar Sáez-Freire, Adrián Blanco-Gómez, Sonia Castillo-Lluva, Aurora Gómez-Vecino, Julie Milena Galvis-Jiménez, Carmen Martín-Seisdedos, María Isidoro-García, Lourdes Hontecillas-Prieto, María Begoña García-Cenador, Francisco Javier García-Criado, María Carmen Patino-Alonso, Purificación Galindo-Villardón, Jian-Hua Mao, Carlos Prieto, Andrés Castellanos-Martín, Lars Kaderali, Jesús Pérez-Losada

**Affiliations:** a Instituto de Biología Molecular y Celular del Cáncer (IBMCC-CIC). Universidad de Salamanca/CSIC. Salamanca, Spain; b Instituto de Investigación Biosanitaria de Salamanca (IBSAL). Salamanca, Spain; c Departamento de Fisiología y Farmacología. Universidad de Salamanca. Salamanca. Spain; d Departamento de Bioquímica y Biología Molecular I. Facultad de Biología. Universidad Complutense de Madrid. Madrid, Spain; e Instituto Nacional de Cancerología, Bogotá, D.C., Colombia; f Servicio de Bioquímica Clínica. Hospital Universitario de Salamanca. Salamanca, Spain; g Departamento de Cirugía. Universidad de Salamanca. Salamanca. Spain; h Departamento de Estadística. Universidad de Salamanca. Spain; i Environmental Genomics and Systems Biology Division, Lawrence Berkeley National Laboratory, Berkeley, California 94720, USA; j Bioinformatics Service, Nucleus, University of Salamanca (USAL), Salamanca, Spain; k Institute for Bioinformatics. University Medicine Greifswald. Greifswald, Germany

## Abstract

The data presented in this article are related to the research paper entitled “The biological age linked to oxidative stress modifies breast cancer aggressiveness” (M.M. Sáez-Freire, A. Blanco-Gómez, S. Castillo-Lluva, A. Gómez-Vecino, J.M. Galvis-Jiménez, C. Martín-Seisdedos, M. Isidoro-García, L. Hontecillas-Prieto, M.B. García-Cenador, F.J. García-Criado, M.C. Patino-Alonso, P. Galindo-Villardón, J.H. Mao, C. Prieto, A. Castellanos-Martín, L. Kaderali, J. Pérez-Losada). The data shown were obtained from a population of transgenic mice, MMTV-*Erbb2/Neu*, with different susceptibility to breast cancer and a mixed genetic background generated by backcrossing. It was observed that the aggressiveness of breast cancer negatively correlates with age, being lower in chronologically old mice, similar to what occurs in humans. Given that oxidative stress is associated with tumour susceptibility and the degree of aging, the association between the aggressiveness of breast cancer and multiple intermediate phenotypes directly or indirectly related to oxidative stress was studied. Using a mathematical model, we defined biological age and the degree of aging as the difference between biological and chronological ages. As a result, we observed that biologically old mice predominated among those that developed the disease early on, that is, those that were chronologically young. We then identified the specific and common genetic components of Quantitative Trait loci or QTL associated with different evolution of breast cancer, the intermediate phenotypes related to oxidative stress studied, the biological age and the degree of aging. Lastly, we showed that the expression pattern in the livers of biologically old mice were enriched in signalling pathways related to inflammation and response to infections; whereas the biologically young mice exhibited enriched pathways related to mitochondrial activity. For the explanation and discussion of these data refer to the research article cited above.

**Specifications table**

**Table:** 

Subject area	Health sciences.
More specific subject area	Oxidative stress, aging and breast cancer.
Type of data	Text file, Tables, Figures.
How data was acquired	Mice monitorization, Histopathology, ELISA, Fluorometric detection, Mass spec, Expression arrays, QPCR, Modular clinical chemistry analyser, DNA genotyping, Linkage analysis.
Data format	Analysed.
Experimental factors	Heterogeneous cohort of mice generated by a backcross between FVB and C57 genetic backgrounds. FVB carries the MMTV-ErbB2/Neu transgene.
Experimental features	The mice were monitored weekly and at the end of their lives were evaluated by necropsy and histopathology. The signalling molecules and the parameters directly or indirectly related to oxidative stress were evaluated by different ELISA techniques. Telomeric length was determined by QPCR. Gene expression was evaluated using expression arrays. The metabolites were determined using mass spectrometry and a clinical chemistry analyser.
Data source location	Instituto de Biología Molecular y Celular del Cáncer (IBMCC-CIC). Universidad de Salamanca/CSIC. Salamanca, Spain; and Institute for Bioinformatics. University Medicine Greifswald. Greifswald, Germany.
Data accessibility	The data are available with this article. Gene expression data for mouse livers are available through Gene Expression Omnibus GSE 99962.

**Value of the data**

• These data show that in a genetically heterogeneous mouse population breast cancer is more aggressive in young mice, similar to occurs in humans.
• We identified multiple intermediate phenotypes directly or indirectly related to oxidative stress, associated with the aggressiveness of breast cancer and with the chronological age.
• We identified the quantitative genetic component associated with the aggressiveness of breast cancer, aging and different intermediate phenotypes of oxidative stress.
• We proposed a mathematical model to define biologically young and old mice based on oxidative stress parameters.
• A similar strategy could be used in humans to define the biological age based on oxidative stress or other parameters.

## 1 Data

**Fig. 1:**
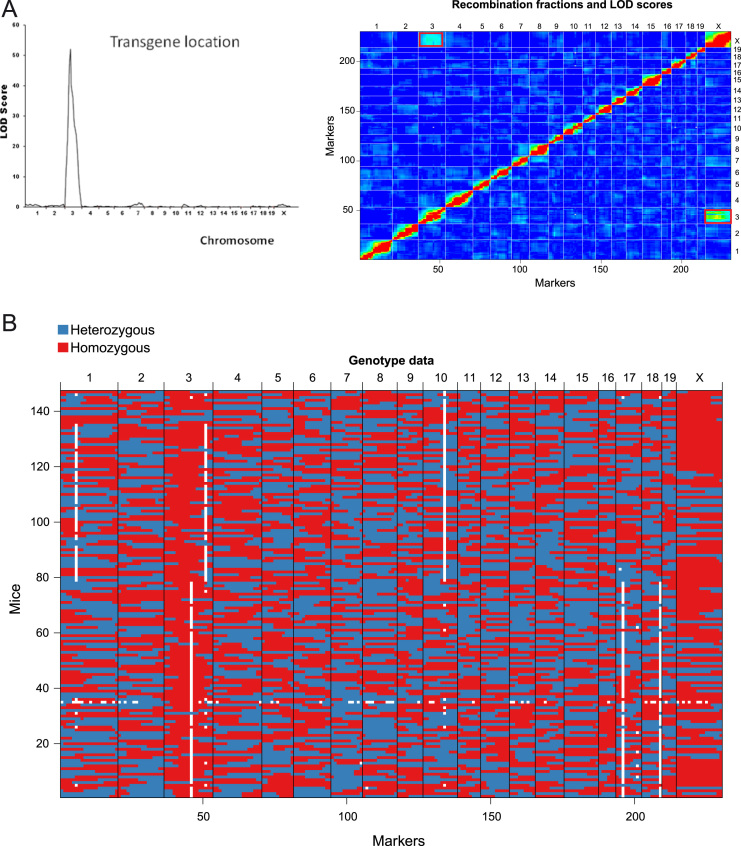
MMTV*-ErbB2/Neu* transgene insertion and genetic background of the backcross cohort of mice. **A**) The MMTV-*ErbB2/Neu* transgene is inserted on chromosome 3, as shown by the linkage analysis. On the right, the heatmap shows the estimated recombination fractions (upper left) and LOD scores (lower right) for all pairs of genotyped markers in the backcross population. Red indicates pairs of markers that appear to be linked (low recombination fraction or high LOD), and blue indicates pairs that are not linked (high recombination fraction or low LOD). Red rectangles indicate low recombination fraction (or high LOD) between markers on chromosome 3 and chromosome X. The apparent linkage between markers on these chromosomes was due to the selection of female transgenic backcross mice for this study, and it shows the transgene is located on chromosome 3. **B**) Genotype data of the 147 backcross mice at the 244 SNPs. Red and blue spots correspond to homozygous and heterozygous genotypes, respectively. White spots indicate missing genotype data. Black vertical lines indicate the boundaries between chromosomes.

A cohort of mice with heterogeneous susceptibility and evolution to breast cancer was generated, and different pathophenotypes of the disease were evaluated (Supplementary Table 1). To generate the cohort, a backcross was made between mice with the C57 and FVB genetic backgrounds; the latter carries the MMTV-*ErbB2/Neu* transgene [1]. The cohort was genotyped by a panel of SNPs (Supplementary Table 2). The linkage analysis demonstrated that the transgene was integrated on chromosome 3 (Fig. 1**A**). Using this strategy each mouse of the cohort was genetically unique (Fig. 1**B**).

**Fig. 2:**
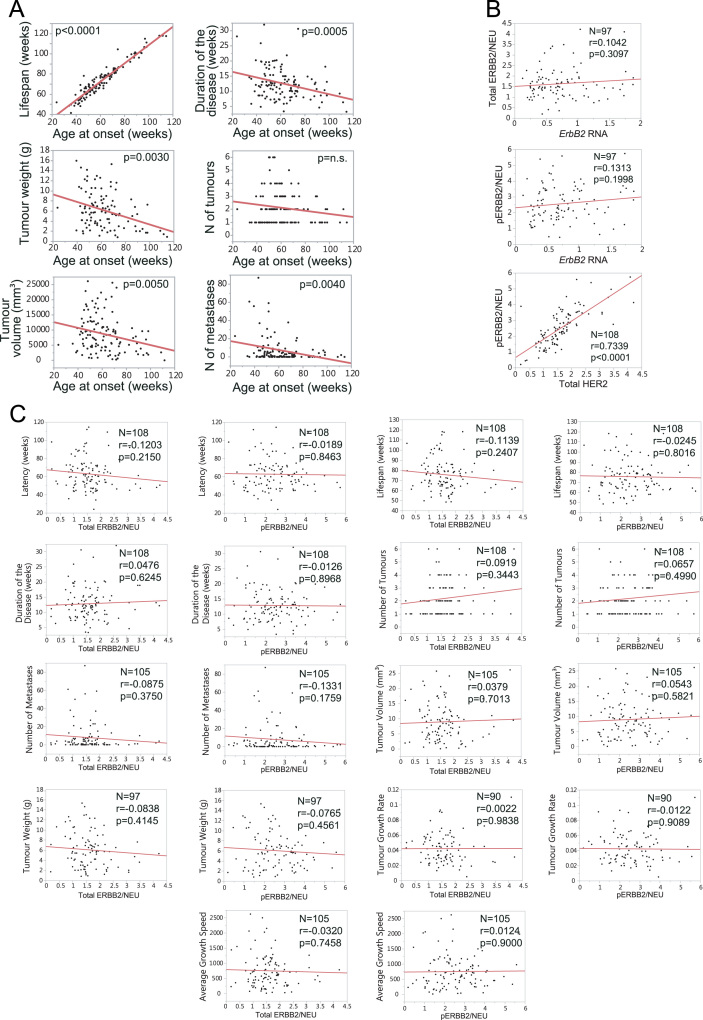
ERBB2 and pathophenotypes of breast cancer variability. **A**) The correlation studies show that the disease was less aggressive in a continuum as age advances. Units: age at onset, lifespan and duration of the disease in weeks; tumour weight in grams; tumour volume in mm^3^. **B**) ErbB2 RNA levels did not correlate with total or phosphorylated ERBB2 protein levels. In contrast, total and phosphorylated ERBB2 protein levels correlated with high statistical significance. **C**) Total and phosphorylated protein levels of lllERBB2 did not correlate with the variability of any of the breast cancer pathophenotypes developed in the backcross population.

We observed that the heterogeneous evolution of breast cancer in the mouse cohort was associated with age. Thus, mice that developed the disease early on had a more aggressive cancer than those that developed it late. This fact was demonstrated by comparing the behaviour of the disease between young versus old mice (*see main paper*). We distinguished between young and old mice according to the median age (79 weeks) (Supplementary Table 1). In addition, a negative correlation was observed between the aggressiveness of different pathophenotypes of breast cancer throughout the age in the study cohort (Fig. 2**A** and Supplementary Table 3). The aggressiveness of the disease did not correlate with the ERBB2/NEU protein levels (Fig. 2**B** and **C** and Supplementary Table 3).

The association between greater aggressiveness of breast cancer at young age and vice versa is also described in humans [2], [3], as well as the association of oxidative stress with both cancer and aging [4], [5], [6]. Oxidative stress, cancer and aging are complex traits that are influenced by multiple intermediate phenotypes [7]. The pathogenic association between these three entities indicates that they share multiple intermediate phenotypes. Therefore, we studied the association between multiple intermediate phenotypes, directly or indirectly related to oxidative stress, with the behaviour of breast cancer and with chronological age (survival) (Supplementary Table 4) in the backcross cohort.

**Fig. 3:**
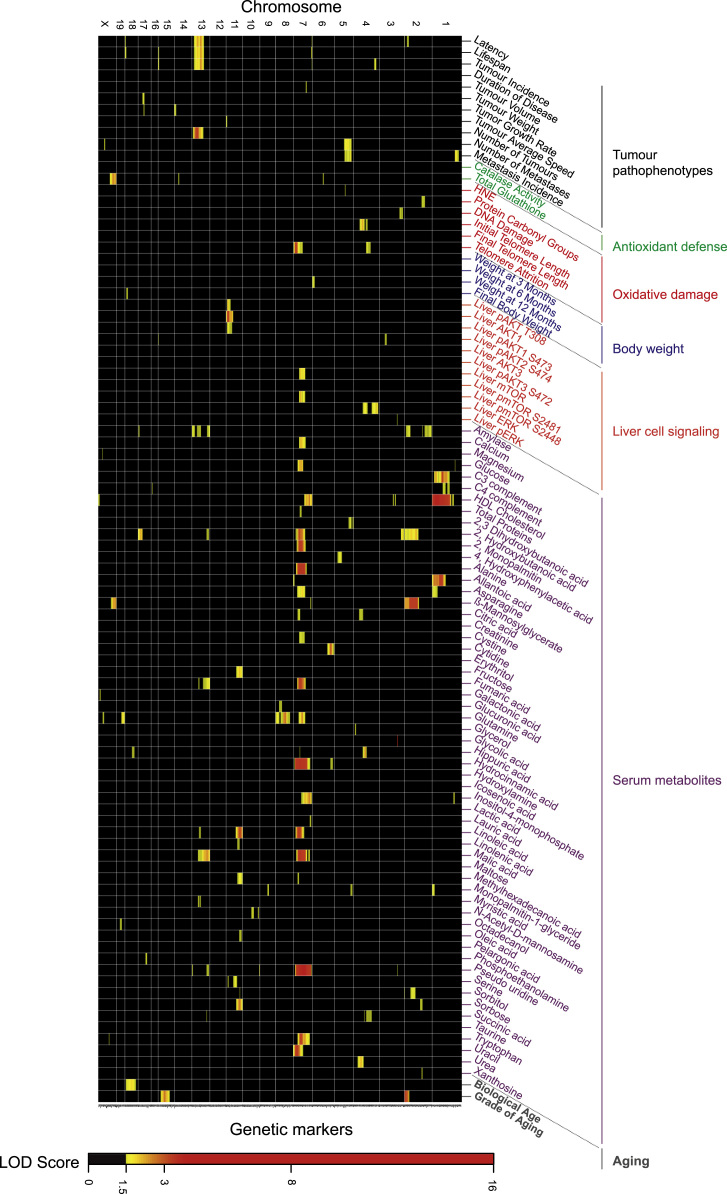
The heatmap shows the specific associations between breast cancer pathophenotypes and intermediate phenotypes.

The association between cancer, aging and oxidative stress indicates that these complex traits must also share multiple quantitative trait loci (QTLs) at the genetic level. These QTLs would control part of the variability of intermediate phenotypes associated with complex traits [7], [8]. Previously, we identified part of the genetic component associated with the variable evolution of breast cancer in the backcross cohort [9]. We have now identified QTLs associated with the different intermediate phenotypes of oxidative stress studied here and represented them together with the QTLs of breast cancer (Fig. 3). The specific data of the identified QTLs are described in Supplementary Tables 5, 6 and 7.

**Fig. 4:**
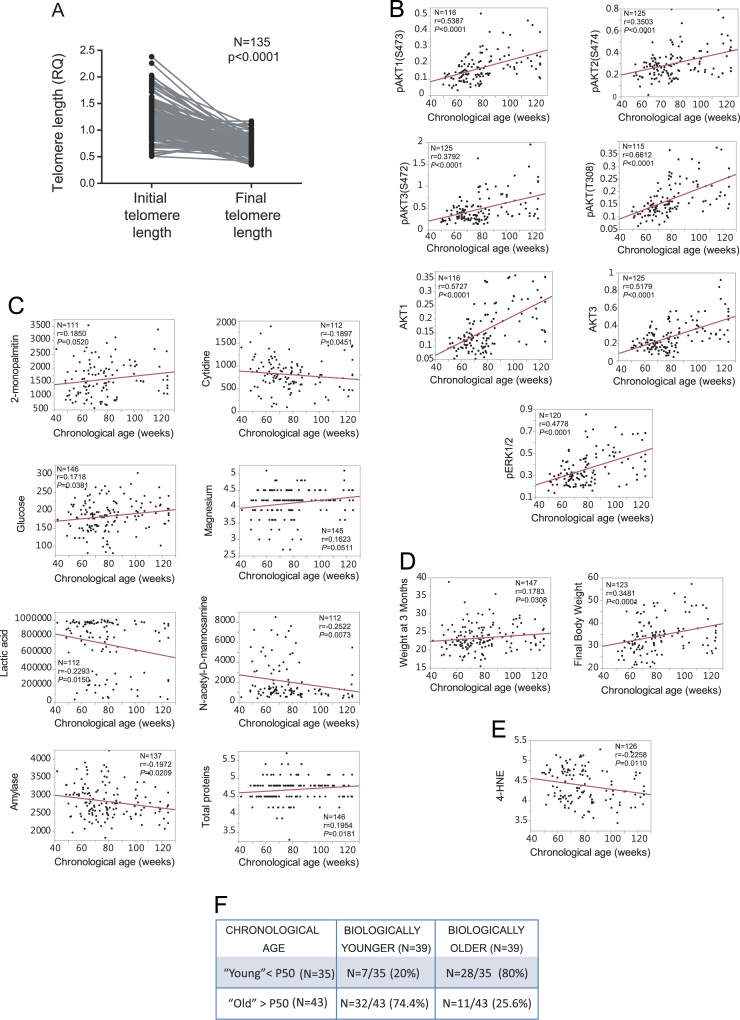
Associations of the chronological age at the time of dead with different subphenotypes related to oxidative stress. **A)** Comparison of the telomere length measured from tail tissue of backcross mice at the initial stage and at the time of death. Paired t-test. **B**) Correlation of the chronological age at the time of death with various signalling molecules determined in the liver by ELISA. **C**) Association of the chronological age with metabolites determined in serum at 3 to 4 months of age in a disease-free stage. **D**) Correlation with weight at four months (weight 1) and weight without tumours after necropsy. **E**) Correlation with the levels of 4-HNE as an indicator of lipid oxidation. **F**) The number of young and old mice based on the biological and the chronological age. The Ns indicate the animals where all of the variables were available and included within the model.

We identified the intermediate phenotypes of oxidative stress that correlated with the chronological age (survival) (Fig. 4**A–E**) and generated a multivariate model to define the biological age. Then, we calculated the degree of aging for each mouse, that is, to define whether it was biologically young or old. For this purpose, the chronological age was subtracted from the biological age predicted by the model (*see main paper*). The number of biologically young and old mice within the cohort is indicated (Fig. 4**F**).

**Fig. 5:**
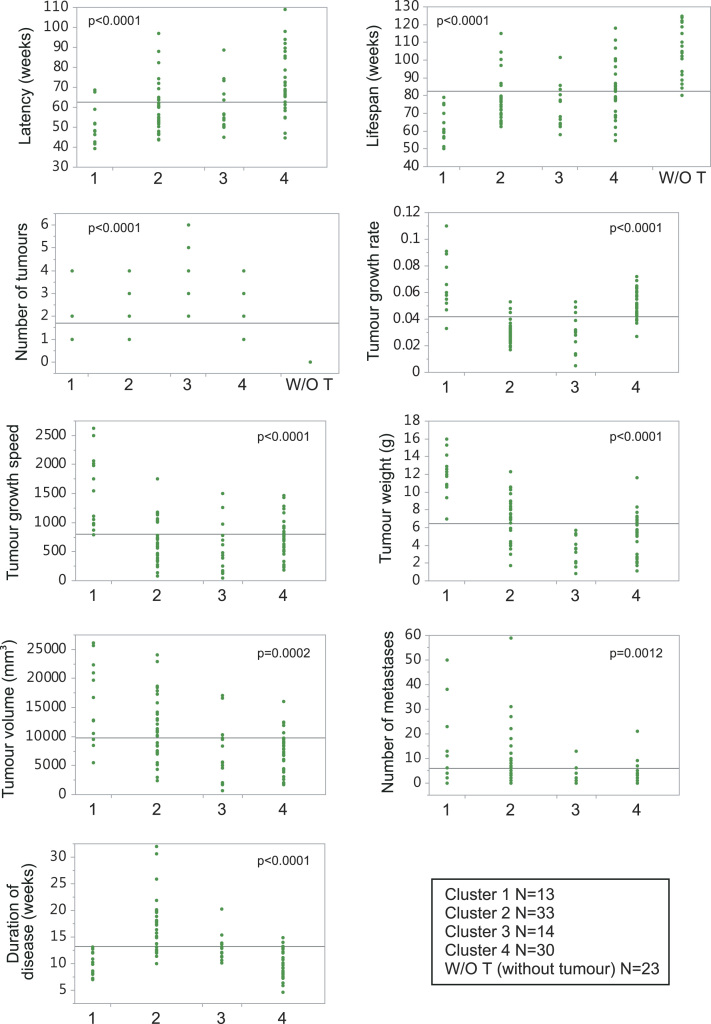
Behaviour of the pathophenotypes of breast cancer in the clusters generated by principal component analysis.

The mice were classified into four clusters by principal component analysis according to the severity of the disease (*see main paper*). The behaviour of each pathophenotypes in the clusters is shown (Fig. 5). We observed that in the clusters of poor evolution (1 and 2) mice chronologically young, but at the same time biologically old, predominated; whereas in clusters of good evolution (3 and 4, and in an additional cluster formed by mice without tumours two years after the end of the experiment) the chronologically old mice predominated, but at the same time were biologically young (*see main paper*).

We identified two QTLs associated with the degree of aging, gaQTL1 and gaQTL2. The expression, determined by expression arrays, of some genes located in gaQTL1 in the liver correlated with the degree of aging. Of them, only *Zbp1*, a gene related to inflammation and response to infection [10], was confirmed by QPCR (Supplementary Table 8).

**Fig. 6:**
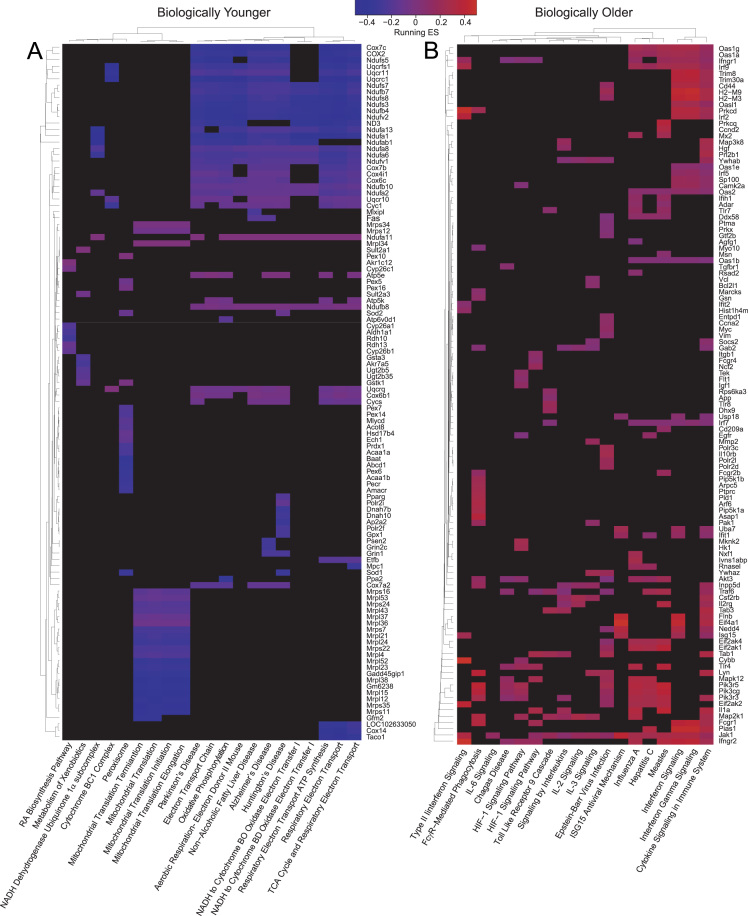
Genes in enriched pathways identified by GSEA. The heatmaps represent the running enrichment score of the genes with absolute value of Rank Metric Score >2.0 that mainly accounted for the enrichment signal found in some of the gene sets differentially enriched in biologically younger and biologically older mice. The left heatmap (A) shows the pathways that were enriched in biologically younger mice (ES < 0) and the right heatmap (B) shows the enriched pathways in biologically older mice (ES > 0).

Next, the global gene expression was analysed in mice livers and it was observed that the expression patterns in the biologically old mice were enriched in genes related to inflammation and the response to infection (Fig. 6 and Supplementary Table 9A). Thus, it is possible that Zbp1 could be one of the genes with a greater effect, since it was already detected at the genetic level as putative responsible of gaQTL1 effect. On the other hand, expression patterns in biologically young mice were enriched in genes related to mitochondrial function (Fig. 6 and Supplementary Table 9B).

## 2 Experimental design, materials and methods

### 2.1 Tumour pathophenotypes

Female transgene-positive mice were monitored and palpated once a week for the manifestation of primary mammary tumours. The temporal stages of evolution and tumour progression of the traits of the ERBB2-positive disease were differentiated [9]. The following variables related to the *temporal stages of disease evolution* were distinguished: (i) *tumour latency*: the period between the date of birth and the age when the first mammary tumour was palpated. (ii) *Duration of the disease*: period between the appearance of the first tumour and the moment when the mouse was euthanized; and (iii) *lifespan*: the sum of tumour latency and duration of the disease. In addition, the following *local tumour progression traits* were considered: *number of tumours* - all visible tumours at necropsy; and *local tumour progression parameters*. These parameters included: (i) tumour volume, estimated every week using the formula [11]:

$$\mathrm{Tumour}\;\mathrm{volume}=\frac{\mathrm{length}\times {\mathrm{width}}^{2}}{2}$$

(ii) Average growth speed, obtained using the expression:

$$\mathrm{Average growth speed}=\frac{\left(\mathrm{Final}\;\mathrm{Volume}–\mathrm{Initial}\;\mathrm{Volume}\right)}{\mathrm{Duration}\;\mathrm{of}\;\mathrm{disease}}$$

(iii) Tumour growth rate was obtained after the logarithmically transformation of the data and the estimation of linear regression for each tumour; and (iv) final tumour weight was determined at the time of necropsy. Lastly, we also evaluated *distant tumour progression*, where the incidence and number of lung metastasis at the time of necropsy was quantified. The mice were euthanized when they showed signs of sickness or when the tumours reached 2.5 cm in diameter.

### 2.2 Quantification of the liver signalling pathways associated with pro-oxidant activity

In brief, the livers were collected at necropsy, snap-frozen in liquid nitrogen and kept at −80 °C. Proteins were extracted from frozen tissues. Ceramic beads, from the Precddellys Lysing Kit CkMix, (Precellys, Bertin Technologies, catalogue number 03961-1-009, Montigny le Bretonneux, France) were added to the tissues (10 to 50 mg). Tissues were homogenized for 10 s, 5.5 m/s (twice), using FastPrep Homogenizer (Thermo Savant, Thermo Fisher Scientific Inc., Waltham, MA USA) in 2× Cell Lysis Buffer containing protease inhibitors (Cell Signalling, catalogue number 9803, Danvers, MA, USA) and 2 mM phenylmethylsulfonyl fluoride (PMSF). Samples were incubated for 20 min on ice, and protein extracts were passed through QIAshredder homogenizer columns (Qiagen, catalogue number 79656, Hilden, Germany) to break down DNA. Supernatants were collected and quantified using the BCA Protein Assay Kit (Thermo Fisher Scientific Inc., catalogue number 23228, Waltham, MA USA) and Albumin Standard (Thermo Fisher Scientific Inc., catalogue number 23209, Waltham, MA USA).

Levels of phosphorylated and total AKT2, AKT3, mTOR and total ERK were measured using the Sandwich ELISA Kit (Pathscan Cell Signaling Technology, Danvers, MA, USA) and phospho-AKT2 (Ser474) (catalogue number 7932), total AKT2 (catalogue number 7930, phospho-AKT3 (Ser472) (catalogue number 7942), total AKT3 (catalogue number 7934), phospho-mTOR (Ser2481) (catalogue number 7978), phospho-mTOR (Ser2448) (catalogue number 7976, total mTOR (catalogue number 7974) and total p44/42 MAPK (ERK1/2) (catalogue number 7050). The levels of phosphorylated and total AKT1 and phosphorylated ERK were measured with the Sandwich ELISA Antibody Pair (BD Laboratories, 353077, San Jose, CA, USA) and the capture antibody (Pathscan Cell Signaling Technology). In the same manner, phospho-Akt (Thr308) (catalogue number 7144), phospho-AKT1 (Ser473) (catalogue number 7143), total AKT1 (catalogue number 7142) and phospho-P44/42 MAPK (Thr202/Tyr204) (catalogue number 7246) were also quantified.

### 2.3 Quantification of telomere length

*Telomere length* in breast tumours and normal tissues (tail) was quantified by QPCR. Telomere length from tail tissue was measured when the animals were three months old (initial telomere length), and again after the animals were euthanized (final telomere length). This allowed telomere attrition to be determined by subtracting the initial telomere length from the final telomere length and dividing by the time between the two time points. QPCR was performed following the conditions published elsewhere [12]. Briefly, PCR reactions were carried out in a total volume of 12 µl in *Twin-Tec real-time PCR plates 96 (#0030132513, Eppendorf)* with the following reagents: 6 µl of *PerfeCTa SYBR*® *Green SuperMix ROX #733–1188, VWR (1X);* 1.6 µl of 150 mM forward and reverse telomeric primers (*5′- CGGTTTGTTTGGGTTTGGGTTTGGGTTTGGGTTTGGGTT-3′ and 5′-GCTTGCCTTACCCTTACCCTTACCCTTACCCTTACCCT-3′,* respectively). Also, the acidic ribosomal phosphoprotein PO (36B4) gene was used to normalization the amount of DNA. Forward and reverse primers for 36B4 were *5′- ACTGGTCTAGGACCCGAGAAG-3′ and 5′-TCAATGGTGCCTCTGGAGATT-3′,* respectively; we also added 1 µl of DNA (10ng), and 3.4 μl double-distilled H2O. An automated thermocycler (*Mastercycler ep Realplex2, Eppendorf*) was used to carry out the reactions. The data analysis was done using the *2*^*−ΔΔCt*^ *method* [13].

### 2.4 Statistical analysis

The Pearson or the Spearman test for correlation analysis was performed, depending on the distribution of the data (Kolmogorov-Smirnov test). The t-test or the *U of* Mann Withney was used for comparing two groups and the ANOVA or the Kruskal-Wallis test was used when comparing more than two groups. We used the Kaplan-Meier estimator when comparing temporal variables.

### 2.5 SNP genotyping

SNP genotyping was described previously [9]. Briefly, tail DNA concentrations were measured with a Nanodrop ND-1000 Spectrophotometer and PicoGreen double-stranded quantification (Molecular Probes, Thermo Fisher Scientific Inc., Waltham, MA USA), and were used for genotyping. The genome-wide scan was carried out at the Spanish National Centre of Genotyping (CeGEN) at the Centro Nacional de Investigaciones Oncológicas (CNIO, Madrid, Spain). Illumina's Mouse Low Density Linkage Panel Assay was used to genotype 147 F1BX mice at 377 SNPs. Genotypes were classified as FVB/FVB (F/F) or FVB/C57BL/6 (F/B). Ultimately, 250 SNPs were informative among the FVB and C57BL/6 mice; the average genomic distance between these SNPs was 9.9 Mb. The genotype proportion among the F1BX mice showed a normal distribution.

### 2.6 Linkage analysis

Linkage analysis was carried out using the Interval mapping with the expectation maximization (EM) algorithm and the R/QTL software. The criteria for significant and suggestive linkages for single markers was chosen from Lander and Kruglyak [14]. In QTL results report tables, the markers named as cXX.loc.XX do not refer to real SNP, but to genetic locations where the conditional genotype probabilities for EM algorithm were calculated using the *calc.genoprob* function in R/QTL with step = 2.5 and error.prob = 0.001. To develop multiple QTL models, the *fitQTL* function with Haley-Knott regression in R/QTL was used to fit and compare the models based on the LOD score, and the percentage of variance explained [15]. Only those QTLs with significant additive or an interacting effect, as determined by the “drop-one-QTL-at-a-time” analysis which evaluates the impact of single QTLs or interactions, were included in the model. All the genetic markers used in the linkage analysis are shown (Supplementary Table 2).
